# Investigation of the Superlattice Phases Formed in Ta_72_Ru_28_

**DOI:** 10.3390/ma16020720

**Published:** 2023-01-11

**Authors:** Alexander W. Carruthers, Bradley A. Young, Ed J. Pickering

**Affiliations:** 1 Department of Materials, University of Manchester, Manchester M13 9PL, UK; 2 Department of Materials, University of Oxford, Oxford OX2 6HT, UK

**Keywords:** tantalum, refractory superalloy, ruthenium, coherent intermetalics

## Abstract

The Ta-Ru binary phase diagram has not been fully investigated, but shows potential for a two-phase region of A2 + B2. Given the high melting points of both Ta and Ru, such an alloy would have the potential for high temperature strength. A Ta_72_Ru_28_ alloy was arc melted and investigated in the as-cast and aged (at 1000 °C) states. The as cast alloy was composed of A2 and B2, albeit not in a superalloy-like morphology. A third phase was found in the aged alloy, which has not been reported before, and which is also a coherent superlattice phase of the Ta BCC matrix. The structure of this phase was found to be consistent with the tetragonal Cr_2_Al prototype structure, with lattice parameters of (*a*, *a*, 3*a*), where *a* is the Ta BCC lattice parameter.

## 1. Introduction

There is increasing demand for alloys able to operate at temperatures above which Ni based superalloys can operate. Owing to its higher melting point, tantalum has seen interest as a potential element to base higher temperature body-centred cubic (BCC, A2) superalloys around [1,2,3]. An unconventional alloying element, ruthenium, has been identified as a potential alloying element to form a Ta based superalloy. The rationale for which is discussed below.

### Ta-Ru Binary System

The Ta-Ru binary system has received significant attention as a shape memory alloy (alongside the Nb-Ru system) [4,5,6]. This is due to the martensitic transformation that occurs in near equiatomic Ta-Ru alloys. Comparatively little attention has been given to the alloy system for Ta-rich compositions. A recent modelling based study claimed that a lack of experimental investigation into the Ta-Ru binary remains a significant barrier to accurately modelling it [7].

**Figure 1:**
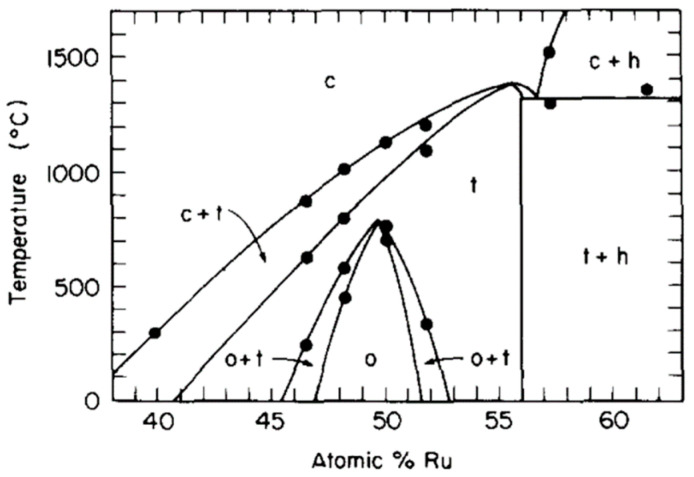
Partial phase diagram of the Ta-Ru system, reproduced from ref. [8].

A detailed phase diagram for near equiatomic compositions was experimentally measured by Chen and Franzen [8], shown in Figure 1. The diagram shows that at such concentrations, hexagonal (h), cubic (c), tetragonal (t) and orthorhombic (o) phases may be formed dependent upon temperature. To note, the phase labelled as orthorhombic in Figure 1 was later shown to be monoclinic [6]. For the purposes of this study, what is interesting to note is that the phase in the Ta-rich, high temperature region of this partial phase diagram is simply labelled as ‘cubic’. The ‘cubic’ phase is based on a BCC structure, and typically labelled as B2 in the studies that have looked at it [8,9]. (100) type reflections can be seen in the XRD trace in ref [8], indicating a B2 structure is present.

Pure Ta is BCC (A2) at all temperatures up to its melting point, and to the authors knowledge at the outset of the study, there were no other phases, other than those listed above, in the Ta-rich half of the Ta-Ru binary phase diagram. Thus, there may exist a two-phase region in the phase diagram where both an A2 and a B2 structure are stable. Such a combination has the potential to form a super-alloy like microstructure, with coherent superlattice B2 precipitates dispersed in a BCC matrix. The aim of this paper is to obtain such a microstructure. In order to do this, past literature was examined to narrow the range of potential compositions. It is interesting to note that very few micrographs were found in the literature, with the alloy phases mainly being identified with diffraction techniques. This leaves the possibility that previously observed alloys may have contained both B2 and A2 structures, given the similarity in diffraction data obtained from such a two-phase microstructure and a purely B2 microstructure.

Formation of the tetragonal phase on the high Ru side of the phase diagram should be avoided. The minimum Ru content in which the tetragonal phase can form appears to be around 40 at% Ru. Some tetragonal phase was found in a 40 at% Ru in one study [8], but not in another [9]. This may be attributable to differences in cooling rates of the alloys. No tetragonal phase was observed in a 37.5 at% Ru alloy [10]. On the low Ru side of the diagram, Hartley et al. [11] examined Ta-Ru alloys aged at 1500 °C and quenched. They found the solubility of Ru in Ta to be between 20 and 30 at% Ru, with a B2 phase being present in the 30 at% Ru alloy. An A2 phase was found in a 25 at% Ru alloy after heating to 1600 °C [12]. It therefore seems reasonable to assume that the onset of B2 formation (at least for some temperatures) lies between 25 and 30 at% Ru. For this study, a composition of 28 at% Ru was selected as a reasonable estimate for intersecting the A2 + B2 region of the phase diagram.

Whilst Ru, as a platinum group metal, has very good corrosion resistance properties, its price would likely limit it to niche applications, either very small items, or ‘money no object’ components. However, Ru is being used in GenIV Ni based superalloys [13,14] (admittedly in much lower quantities), and future work on additions of other alloying elements may be able to reduce the required quantities of Ru.

## 2. Experimental

High purity (>99.95%) Ta and Ru were vacuum arc melted into a ~25 g ingot. The ingot was flipped and re-melted 5 times. A weight change of <1% was measured after casting. Following casting, a section of the material was aged at 1000 °C for 340 h in an Ar atmosphere. This temperature is below the typical solution annealing temperatures for refractory alloys. However, this was deliberate. It was expected that there would be dendritic microsegregation within the ‘as cast’ alloy, owing to the disparate melting temperatures of Ta (3020 °C) and Ru (2250 °C). It was hoped this low temperature heat treatment would create a continuous variation of composition in places (the result of Scheil-type solidification), on a sufficiently small scale (<1 µm) that the segregated regions could be considered to be separate alloys of different composition for the sake of this study. Thus, a single alloy could be used to analyse a range of compositional space.

SEM characterisation of the materials was conducted using a Zeiss Merlin with Oxford Instruments X-max extreme EDX and EBSD detectors, Oxford UK. Samples were mechanically polished to an OPS finish. SEM and EDX maps were collected using a voltage of 5 kV for finer resolution. EBSD maps were collected using a voltage of 20 kV. an FEI Scios (FIB/FEG-SEM), equipped with an easylift^TM^ micromanipulator(FEI easylift micromanipulator, Hillsboro, OR, USA) was used to extract electron transparent lamellae. These were prepared for TEM analysis using the focused Ga^+^ ion beam in situ lift-out method [15] using an energy of 5 keV for the final polish.

The lamellae were analysed using a 200 keV, X-FEG FEI Talos F200 S/TEM(200 keV, X-FEG FEI Talos F200 S/TEM, Hillsboro, OR, USA). S/TEM-EDX data were quantified and are presented in at%. The Cliff-Lorimer method was used for quantification. 4D-STEM datasets were acquired with a quantum detectors direct electron Merlin camera(Merlin camera, Oxford, UK). A 10 µm condenser aperture was used to form a ‘pencil beam’. Virtual dark field (DFs) were constructed by integrating the intensities from reflections solely pertaining to the phases of interest.

## 3. Results and Discussion

### 3.1. SEM

**Figure 2:**
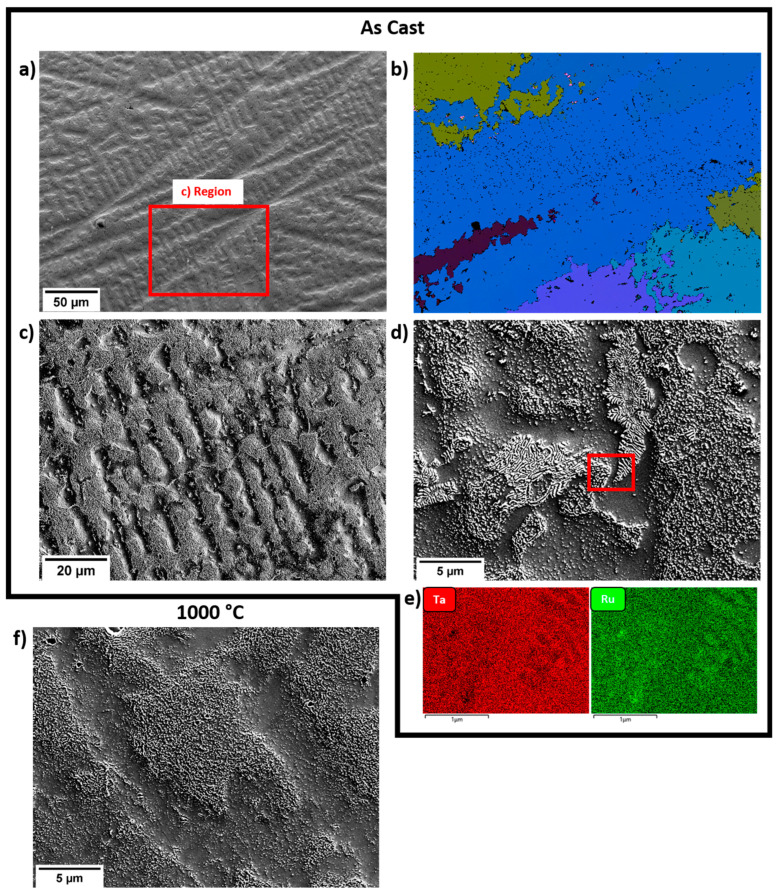
(**a**) 20 kV Secondary electron SEM micrograph of the as cast Ta_72_Ru_28_ alloy and (**b**) corresponding EBSD euler map. All points were indexed as BCC. (**c**,**d**) show higher resolution secondary electron images of the as cast alloy. (**e**) Low voltage (5 keV) EDX maps from the red box in (**d**) showing qualitatively more Ru in the white regions. (**f**) Secondary electron SEM micrograph of the aged Ta_72_Ru_28_ alloys, showing the similar distribution of regions as the as cast alloy.

Secondary electron micrographs, shown in Figure 2, show both the as-cast and aged material had a similar microstructure, which was expected given the relatively low aging temperature (likely to be a homologous temperature of around 0.5 *T*_m_). The materials were composed of a dendritic microstructure containing two phases: a dark phase containing reduced quantities of Ru, and a white phase containing increased quantities of Ru. Both phases are present in both the dendritic and interdendritic regions, but with different proportions. The primary dendritic region is the darker region, richer in Ta, since this has a higher melting point than Ru. This dendritic region only comprises ~30% of the material.

In the interdendritic regions where the concentrations of the white phase were higher, the morphology appeared eutectic in the as-cast material, although this was not confirmed with further experiments. The lamellae in these regions appeared to be broken up by the heat treatment in the aged material. An EBSD scan identified the entire alloy as BCC phase in both the as-cast and heat-treated states. However, it must be noted that EBSD is unable to differentiate between BCC and B2 phases.

SEM-EDX of the area shown in Figure 2a found an average Ru content of 27.3 ± 0.5 at%, which is close to the desired composition. The average Ru content of the primary and secondary dendrites was measured as 22.5 and 29.4 ± 0.5 at%, respectively.

### 3.2. TEM

**Figure 3:**
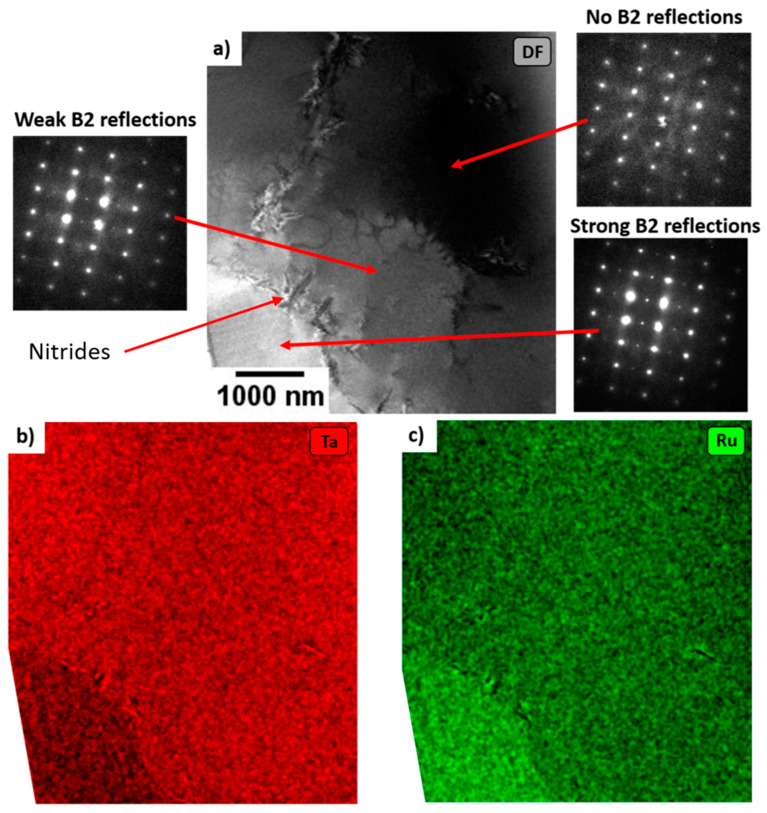
STEM analysis of the as cast material. (**a**) Dark field composed using (100) B2 type reflections, viewed down the [011] zone axis and (**b**,**c**) EDX elemental maps showing a Ru enriched region at the bottom left and small Ru fluctuations across the rest of the foil (a Ru-poor region). Strong B2 reflections can be seen in the Ru enriched region, and weaker B2 reflections can still be seen in the matrix where the Ru content is slightly higher than other areas.

**Figure 4:**
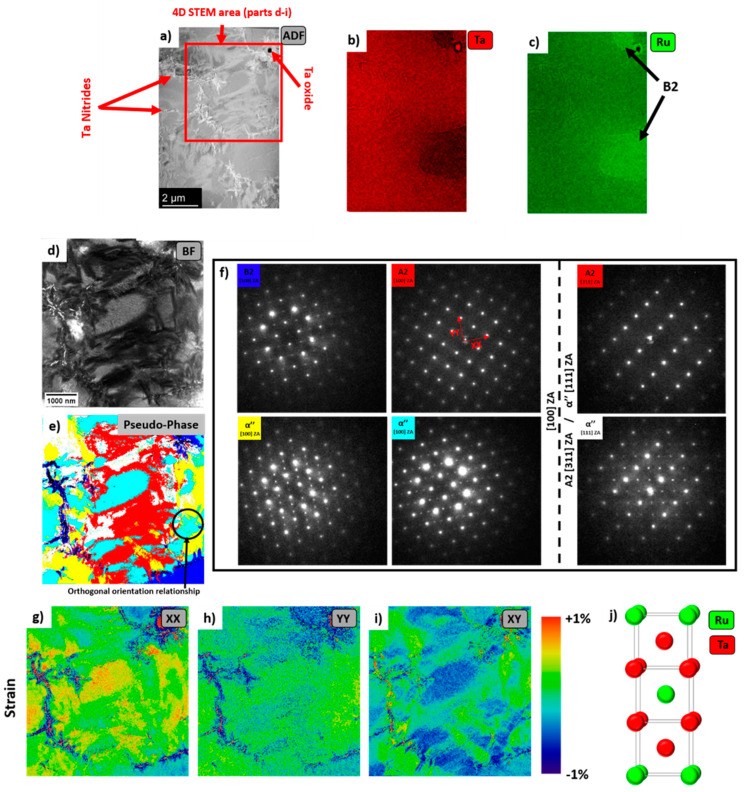
TEM analysis of the aged Ta_72_Ru_28_ alloy. (**a**) ADF and (**b**,**c**) elemental maps. (**d**) virtual BF produced from 4D pencil beam STEM, (**e**) pseudo phase map of the region shown in (**d**), representative diffraction patterns for each colour are shown in (**f**). Blue = B2, yellow, cyan and white = α’’ and red = A2. (**g**,**h**) normal and (**i**) shear strain maps relative to the A2 phase of the region shown in (**d**), the reference directions are shown in (**f**). The XX and YY directions are parallel to the c axis in the cyan and yellow orientations of α’’, respectively. (**j**) the proposed crystal structure of the α’’ phase.

FIB lamellae were extracted from both the as cast and aged samples, the results of which are shown in Figure 3 and Figure 4, respectively. In both cases, the lamellae were taken from across the boundary between the dendritic and interdendritic regions. It was ensured that both lamella contained both the light and dark phases. Electron diffraction confirmed that, in both samples, the Ru-richer (seen as lighter in SEM) phase was indeed B2. However, as can be seen in both the TEM and SEM images, these B2 precipitates are much coarser than would be typically required in a superalloy. Further to this, the Ta-richer (seen as darker in SEM) phase was more complex in both materials.

### 3.3. As Cast

In the as cast sample, Figure 3, the Ru poorer regions were not entirely homogenous, with compositional variations of ~2 at% Ru. In the Ru richer regions, (B2) ordering could be seen. The intensity of the ordered (100) reflection reduced with reducing Ru content. The compositional window over which the A2 and B2 phases are simultaneously stable can therefore be considered to be narrow.

### 3.4. Aged at 1000 °C

Within the aged lamellae, a third phase was identified, referred to hereafter as α’’. To the authors knowledge, the α’’ phase has not been previously reported in the literature for Ta-Ru. The formation of a third phase within a binary alloy appears to break the ‘phase rule’. This cannot be explained by the uptake of oxygen or nitrogen within the alloy. Several Ta nitride and Ta oxide phases were also present in both lamellae due to air contamination during melting, these are labelled. Rather this is evidence that 1000 °C provided insufficient kinetics to cause dissolution of the B2 into the A2 phase. The A2 and B2 phases can be thought of as separate alloys at 1000 °C, as evidenced by no observable change in the microstructure seen in Figure 2. Thus it is the A2 phase alone that has devolved into A2 + α’’.

The α’’ phase was characterised by two extra diffraction spots between the transmitted beam and the reflections from the (002) planes, suggesting a unit cell of dimensions similar to (*a*, *a*, 3*a*), where a is the lattice parameter of the A2 phase. Diffraction patterns of α’’ can be seen in Figure 4f. The α’’ was indexed as the phase prototype Cr_2_Al, space group 139. The proposed structure is shown in Figure 4j. Note that this phase is different from the tetragonal phase reported in higher Ru content (~40 at%) alloys [8]. The α’’ prime phase is named as such here because of the similarities it possesses with that of γ’’ in Ni based superalloys. α’’ is likely stable only at lower temperatures, as the literature, which used higher aging temperatures for this alloy system, does not report its presence. The unit cell is ~3 times, as opposed to γ’’s ~2 times, the length of the matrix phase.

Within both lamellae, with the exception of some slight bending and the oxide/nitride phases, all the phases were fully coherent with each other.

The pseudo-phase map in Figure 4e, was produced by creating virtual dark field images from the reflections of the B2 and two orientations of the α’’ phases. When the brightness of the dark fields fell below an arbitrary intensity, the pixel was considered either A2 or the third orientation of the α’’ orientated with the c direction parallel to the beam direction. In this orientation, the α’’ phase should be indistinguishable from A2. To distinguish between A2 and the 3rd orthogonal orientation of α’’, a second data set was acquired after the sample was tilted to the [311] zone axis for A2/B2 phases. This is equivalent to the [111] direction of the 3rd orthogonal orientation of the α’’ phase. Apparent orthogonal interweaving of the different orientations of the α’’ phase is also seen in the pseudo-phase map.

The phase map shows that there is B2 surrounding the oxide and nitrides in the foil. These interstitials are Ru poor, consequently their surroundings are Ru rich, which then stabilises the B2 phase. No compositional differences could be seen between the A2 and α’’ phases.

The 4D-STEM data was used to calculate strain maps, Figure 4g–i, which were measured with reference to an A2 diffraction pattern. These have been included to show the coherency between the A2, B2 and α’’ phases along different crystallographic directions. The XX and YY directions were set parallel/perpendicular to the 001 directions in the A2 crystal and are shown in Figure 4f,g, respectively. Figure 4i shows the corresponding shear component of the strain.

Immediately, note that the scale bar for Figure 4g–i shows that nowhere does the coherency strain between these phases exceed +/−1%. The most significant, positive, strain can be seen in the α’’ phase parallel to the c axis. E.g. the orientation of the α’’ phase coloured cyan in Figure 4e corresponds with a positive strain in Figure 4g, but not Figure 4h. This demonstrates that whilst the b axis of the α’’ phase closely matches the A2 lattice parameter, the c axis is slightly longer than 3 times the lattice parameter of the A2 phase. This coherency strain relative to the A2 phase is less than some of the observed strains in the γ’’ phase in Ni superalloys [16,17]. It may be the case that, given a suitable (very) high-temperature solution treatment followed by quenching and ageing, that an improved distribution of α’’ phase can be obtained, akin to that found in nickel-base superalloys.

Finally, the B2 phase, seen in the top and bottom right hand corners of Figure 4e can be seen to have a negative strain (again deviating from the A2 by <1%). This is present in both the XX and YY directions, which is to be expected given the cubic crystal structure.

## 4. Conclusions

From this work it is not possible to categorically state that B2 and A2 exist together in *equilibrium* within this alloy system. Further, high temperature heat treatments are necessary in order to be definitive. However, equilibrium is not a necessary condition for the creation of a superalloy. What is necessary is the coexistence of a coherent, order-disordered microstructure. This work does show that it is possible to achieve this within the Ta-Ru alloy system at temperatures of at least 1000 °C.

An ‘as cast’ binary Ta_72_Ru_28_ alloy has been shown to form a binary B2 + A2 microstructure, albeit not in a typical superalloy-like morphology.

After aging at 1000 °C, a second ordered phase based off the BCC lattice formed. This phase, which has not been reported before, has a space group 139 and the unit cell lattice parameters of (*a*, *a*, 3*a*) where a is the Ta BCC phase lattice constant. That a new phase was found in this alloy system demonstrates further that the Ta-Ru system has not been fully investigated.

## 5. Future Work

Future research into this system may wish to examine including additions of other alloying elements to (i) reduce density and (ii) increase the range of temperatures and Ru contents over which the A2 + B2 phases are stable together. Specifically, reducing the necessary Ru content to form the B2 phase would be highly desirable, given the very high cost of Ru.

This may be achievable with the additions of elements such as Ti and Al. Both Ti and Al would reduce the alloys density. In addition, the Ti-Ru binary shows a larger compositional window over which a two-phase A2 + B2 microstructure is present [7] and Al may help stabilize the B2 phase; aB2 phase has been found in both the Al-Ru binary [7] and Ta-Al-Ru ternary systems [18].

More heat treatments would be required to: (i) investigate the thermal stability of the α’’ phase, (ii) reduce the size of the B2 phase, e.g., by solution annealing and rapidly quenching, and (iii) establish if a nano-scale cube-on cube morphology, more typical of superalloys can be formed in this system by high-temperature annealing, followed by quenching and ageing.

The origins of the α’’ phase are of interest. It is currently unclear if it is stable or metastable (e.g., martensitic). The phase does not appear to have a strong lenticular microstructure, see Figure 4e. Although, as was noted in the introduction, equi-atomic Ta-Ru exhibits a strong propensity for martensitic transformations [4,5,6].

All the future work above is still early stages for such an alloys development. Substantial mechanical properties and corrosion tests would need to be performed, particularly high temperature corrosion and creep experiments.

## Data Availability

The raw data associated with this work can be accessed via the following link: https://doi.org/10.5281/zenodo.6513574 (accessed on 1 November 2022).
